# Perinatal origins of bronchopulmonary dysplasia—deciphering normal and impaired lung development cell by cell

**DOI:** 10.1186/s40348-023-00158-2

**Published:** 2023-04-18

**Authors:** I. Mižíková, B. Thébaud

**Affiliations:** 1 Experimental Pulmonology, Department of Pediatrics and Adolescent Medicine, Faculty of Medicine and University Hospital Cologne, University of Cologne, Cologne, Germany; 2 Sinclair Centre for Regenerative Medicine, Ottawa Hospital Research Institute, Ottawa, ON Canada; 3 Department of Cellular and Molecular Medicine, University of Ottawa, Ottawa, ON Canada; 4 Department of Pediatrics, Children’s Hospital of Eastern Ontario (CHEO), CHEO Research Institute, University of Ottawa, Ottawa, ON Canada

**Keywords:** BPD, Prematurity, Lung development

## Abstract

Bronchopulmonary dysplasia (BPD) is a multifactorial disease occurring as a consequence of premature birth, as well as antenatal and postnatal injury to the developing lung. BPD morbidity and severity depend on a complex interplay between prenatal and postnatal inflammation, mechanical ventilation, and oxygen therapy as well as associated prematurity-related complications. These initial hits result in ill-explored aberrant immune and reparative response, activation of pro-fibrotic and anti-angiogenic factors, which further perpetuate the injury. Histologically, the disease presents primarily by impaired lung development and an arrest in lung microvascular maturation. Consequently, BPD leads to respiratory complications beyond the neonatal period and may result in premature aging of the lung. While the numerous prenatal and postnatal stimuli contributing to BPD pathogenesis are relatively well known, the specific cell populations driving the injury, as well as underlying mechanisms are still not well understood. Recently, an effort to gain a more detailed insight into the cellular composition of the developing lung and its progenitor populations has unfold. Here, we provide an overview of the current knowledge regarding perinatal origin of BPD and discuss underlying mechanisms, as well as novel approaches to study the perturbed lung development.

## Introduction

Bronchopulmonary dysplasia (BPD) is the most common form of chronic lung disease in children and a leading cause of neonatal morbidity and mortality [1–3]. Since 1999, the disease has been defined by the need for supplemental oxygen at 36 weeks post-menstrual age, and is classified based on its severity, as well as oxygen and respiratory support requirements into three grades: mild, moderate, or severe [4, 5]. While advancements in neonatal care led to improvements in survival of premature infants, the incidence of BPD has not decreased. Not surprisingly, BPD is now less frequent in infants with birth weight > 1200 g, or born after 30 weeks of gestation, but affects extremely premature infants of lower gestational age [4–6].

BPD is a multifactorial disease which occurs predominantly as a consequence of prematurity leading to respiratory distress and consequent treatments in neonatal intensive care units (NICU), including mechanical ventilation (MV) and oxygen supplementation [2, 5]. In addition to the degree of prematurity, additional antenatal and perinatal risk factors include low body weight (LBW), infection, and maternal nutrition [6–8]. Moreover, preeclampsia and intrauterine growth restriction (IUGR) are identified as independent risks factors [6, 9–11]. Recent studies further indicate that prenatal smoke exposure might also contribute to the development of the disease [12, 13]. Finally, BPD may have some hereditary component [14, 15]. While increased prevalence of BPD is typically associated with male sex, in the long term, female patients with a history of BPD might be affected more severely [6, 16, 17].

In the past BPD was associated mostly with aggressive MV in more mature infants [18]. However, advances in ventilation technology, avoidance of MV, and more judicious use of oxygen result in a new histological phenotype characterized less by fibrosis and more by global arrest in alveolar and microvascular development, as well as impaired, and sometimes declining lung function [4, 5]. BPD is also associated with long-term sequelae, which often persist into adolescence or early adulthood, including neurodevelopmental and cognitive changes [19], impaired lung function [2, 20, 21], pulmonary vascular disease [2, 22], and cardiac dysfunction [2, 23]. Impaired immune development results in increased susceptibility to viral infections and higher risk of rehospitalization later in life [1, 4, 6]. The disease is further associated with increased incidence of asthma [21] and early-onset emphysema [24].

BPD constitutes a complex injury to the developing lung with heterogenous pathological features and outcomes, greatly depending on the degree of prematurity, as well as antenatal and postnatal exposures. In the following paragraphs we discuss underlying causes and mechanisms contributing to the development of BPD, as well as novel approaches to study BPD pathogenesis.

## Intrauterine growth restriction, placental dysfunction, and preeclampsia

IUGR is defined as a failure of the fetus to reach its “biologically based potential” [25]. The condition can arise due to anatomical or functional disorders associated with maternal factors, maternal-placental-fetal unit, or genetic abnormalities [26–29]. IUGR often results in malnutrition, LBW, and permanent perturbations in metabolism and development [26, 28]. It is a known cause of prematurity and associated with increased morbidity and mortality [26, 30].

In experimental animal models, IUGR can be induced by various interventions, including uterine artery ligation [31], low protein diet (LPD) [32–34], or heat exposure [9]. Impaired alveolar and vascular formation during postnatal development and/or in adulthood were reported in various IUGR models in rats [32–35] and sheep [9, 36, 37]. IUGR in developing rat pups from LPD-fed mothers is associated with impaired lung development, as evidenced by increased alveolar septal thickness, *Col1a1* expression and extracellular matrix (ECM) deposition at P23 [34]. These changes are preceded by an inhibition of GH/Stat5/IGF-1 signaling during the embryonic phase. Decreased IGF-1 levels were also reported in serum of BPD patients [38]. Notably, IGF-1 was shown to have anti-inflammatory properties, to preserve lung structure, and to prevent right ventricular hypertrophy (RVH) in a rat BPD model [39–41]. IUGR in rats further impaired embryonic VEGF and BMP signaling, and decreased microvascular and ECM formation postnatally [35]. Finally, microRNA microarray analysis revealed perturbations to “tissue repair” and “cellular communication” pathways [33]. In addition to structural changes, IUGR impairs lung function in developing rats [42, 43], while in clinical studies, IUGR and LBW are associated with poorer lung function in childhood [44, 45] and adulthood [46–48]. The impact on lung function may be directly related to a higher prevalence of BPD among the IUGR patients [11]. Finally, studies show that IUGR and LBW may contribute to development of chronical illness such as asthma [49] or chronic pulmonary obstructive disease (COPD) [50, 51].

Placental dysfunction and preeclampsia (PE), major causes of IUGR, also impact lung development. While the underlying molecular mechanisms remain unknown, PE is an independent risk factor for both, preterm delivery, and the development of BPD [6, 10, 52]. Pathological placental changes resulting from maternal vascular underperfusion (MVU) are associated with increased risk of BPD [53]. A recent meta-analysis of 211 studies show that placental vascular dysfunction in association with IUGR or being born small for gestational age (SGA) increases the risk of BPD and pulmonary hypertension (PH) [54]. Accordingly, decreased levels of cord blood angiogenic factors are strong predictors of BPD-associated PH [55]. Taken together, these data strongly indicates the strong association between the placental dysfunction of prematurity and vascular phenotype of BPD, supporting the so-called “vascular hypothesis” of BPD pathogenesis and the potential preventive use of proangiogenic agents in such patients [56].

While several preclinical models of PE have been designed, most differ from the human condition and replicate the condition to only a limited extent [57]. These include genetic models, such as the hypertensive BPH/5 mouse model [58], chronic hypoxia models [59, 60], or pharmacologically-induced models, such as nitric oxide inhibition [61, 62]. Widely used are also surgical models, such as the reduced uterine perfusion pressure (RUPP) [63], or selective RUPP rat model [64]. Interesting is also the CBA/J × DBA/2 J mouse model of recurrent miscarriage and spontaneous PE, recapitulating many features of the clinical condition, including renal damage, placental growth defects, restricted fetal growth, and increase sFLT-1 and leptin levels [65]. However, the animals do not become hypertensive, therefore not meeting the clinical criteria. Building on the notion of the importance of the above-mentioned sFLT-1 is another rat model, where PE is induced by intraamniotic sFLT-1 injections [66]. This model recapitulates retardation in lung growth, as well as findings of abnormal lung function, vessel density, and RVH. Importantly, both antenatal and postnatal treatment with selective anti-sFLT-1 antibody improved alveolarization, vessel formation, and lung function and decreased RVH in developing pups. Moreover, authors have shown similar result of anti-sFLT-1 treatment in the endotoxin-induced rat chorioamnionitis model [66].

Finally, maternal malnutrition or overnutrition are also associated with metabolic changes leading to increased risk of developing diabetes, obesity, and metabolic syndrome in later life [67–69]. Combined insult of IUGR and maternal high-fat diet (HFD) increase the risk of early cardiovascular pathology in rats [31]. Furthermore, HFD in rat dams prior to conception, or HFD diet from pre-conception until lactation increases airway resistance in the offspring [70]. Taken together, these findings highlight the impact of perinatal nutrition on lung development and the origin of adult pulmonary disease.

## Patent ductus arteriosus, pulmonary hypertension, and microbiome

Patent ductus arteriosus (PDA) is a frequent complication in very preterm infants, with up to 70% of infants born before 28th week of gestation requiring pharmacological or surgical treatment [71, 72]. The condition is associated with increased lung blood flow, impaired lung mechanics, oxidative stress, and increased need for MV. PDA is clinically often associated with RDS and BPD and is historically considered a risk factor for BPD [73–77]. However, whether PDA plays a causal role in BPD pathogenesis is not known [72, 78, 79]. In fact, multiple randomized control trials have failed to find a direct relationship between PDA and the development of BPD [78, 80–83]. PDA closure is performed either pharmacologically or surgically. Pharmacological closure, typically achieved with indomethacin or ibuprofen, was shown to improve alveolarization and lung mechanics, as well as the need for ventilator support [84–86]. Whether the improvement is due to PDA closure or the pharmacological agents themselves however is not fully understood. These improvements have not been observed after surgical closure, with some reports indicating that early surgical ligation itself may contribute to impaired alveolarization [78, 87, 88]. Additionally, studies indicate differences in outcome dependent on timing of the pharmacological closure [89, 90]. Consequently, recent studies and review literature recommend avoidance of surgical ligation and further investigations into timing of PDA closure [78, 91, 92].

During the fetal development, gas exchange is provided by placenta and fetal pulmonary vascular resistance (PVR) is high. Perinatal transition is normally marked by a significant decrease in PVR, resulting in up to tenfold increase in pulmonary blood flow [93–95]. However, in some instances this change in vascular resistance does not occur, resulting in PH. Neonatal PH is a frequent complication in premature infants, particularly those with extremely LBW [96]. While not fully understood, among the known risk factors contributing to neonatal PH are low birth weight, SGA status, oligohydramnios, PE, severity of BPD, and prolonged MV [94, 97]. A consensus approach to better classify pediatric PH recognizes 10 categories of pulmonary hypertensive vascular disease, including the BPD-PH category [98, 99]. Up to 25% of patients with moderate to severe BPD also develop PH [99–101]. It can present itself as primary PH, acute PH associated with RDS or chronic PH associated with BPD (BPD-PH). Additionally, neonatal PH can be exacerbated by a PDA. BPD-PH is characterized by aberrant pulmonary vascular growth and remodeling, RV failure, increased mortality, and increased risk of PH and right ventricular dysfunction in adulthood [99, 102]. Initial diagnosis of neonatal PH is typically based on echocardiography and clinical representation. Late onset of PH in BPD patients (3–4 months of age) is now well described and justifies continuous screening for PH in premature infants with BPD [96]. Current therapies are based on the underlying pathophysiology of neonatal PH and include maintaining adequate oxygen saturation, correction of acidosis, surfactant therapy, and the use of pulmonary vasodilators, such as inhaled nitric oxide and sildenafil [94, 95].

Another intriguing, but understudied factor impacting the perinatal period is the microbiome. Studies have revealed alterations in airway and lung microbiome of prematurely born infants and suggest a link between the microbiome and BPD severity. A recent systematic reviews showed that most studies indicate decreased bacterial diversity, higher levels of Ureoplasma, and lower levels of Staphylococcus in the tracheal aspirates (TAs) of preterm infants who went on to develop severe BPD [103–107]. BPD progression was further associated with microbial turnover and relative abundance of different bacterial strains. It is important to note that majority of the infants included in these studies have received prenatal or postnatal antibiotics, which has previously also been associated with increased risk and severity of BPD [103, 105, 108–110]. Finally, a recent study has explored the relationship between perinatal microbiome and metabolome. The authors observed an increase in Proteobacteria, a reduction in Lactobacilli, as well as reduction in fatty acid β-oxidation pathway in infants with BPD [111]. While this data suggests a role for airway microbiome in the regulation of inflammation, further studies are needed to identify the mechanisms by which microbiome at birth modulates and primes the pulmonary metabolome.

## Prenatal inflammation

Increasing evidence suggests that pre- and postnatal inflammation play critical roles in the development of BPD. BPD is associated with an increase in pro-inflammatory and decrease in anti-inflammatory cytokine levels, lung neutrophil and monocyte infiltration, and macrophage activation. Whether pre- or postnatal inflammation contribute more to the development and severity of BPD is not known.

Multiple types of prenatal inflammation have been suggested as BPD risk factors, including chorioamnionitis [112], fetal inflammatory response syndrome [113], and neonatal leukemoid reaction [114]. Most widely studied in the context of BPD is chorioamnionitis, a complex syndrome associated with preterm delivery. Chorioamnionitis is an inflammation of chorion and amnion membranes, often caused by bacterial infection, and usually classified as either histological or clinical [115]. The histological form is characterized microscopically by inflammatory cells infiltration, while the clinical form is defined by abdominal pain, presence of uterine tenderness, fever, increased white blood count, and maternal and fetal tachycardia [115, 116]. Studies in experimental animals suggest that antenatal inflammation can affect the expression of grow factors, modulate the immune system, and contribute to structural changes in the lung, resembling the BPD phenotype [117]. Intrauterine inflammation in animal models is often induced by bacterial lipopolysaccharide (LPS). Pre- or postnatal LPS in rodents and sheep results in inflammation, alveolar hypoplasia, impaired surfactant production, and impaired pulmonary vascular function [118–121]. Importantly, amniotic concentrations of IL-6, IL-8, IL-1β, and TNF-α are increased in mothers of infants who develop BPD [122]. However, the clinical data on relationship between chorioamnionitis and BPD remain inconclusive [123–125]. This is partly due to an inconsistent diagnosis and lack of correlation between clinical and histological chorioamnionitis [116, 126]. In fact, some studies indicate that the increased risk of BPD is rather associated with the postnatal consequences of chorioamnionitis and prematurity, such as surfactant deficiency, neonatal sepsis, and need for MV [112, 126–128]. Additionally, postnatal sepsis directly increases BPD incidence and interrupts lung development by various mechanisms, including inflammation, oxidative stress, and endothelial injury [126, 129].

## Mechanical ventilation, hyperoxia, and postnatal inflammation

Prematurity in infants is furthermore directly associated with the need of respiratory support and ventilation. Ventilation constitutes a major risk factor for lung injury and BPD. Initial injury results from both, barotrauma and volutrauma, which initiate an influx of immune cells, particularly macrophages and neutrophils, and an increase in production of pro-inflammatory cytokines [130–133]. Elevated levels of pro-inflammatory IL-1β, IL-6, and IL-8 are found in TAs and blood of BPD patients [134]. Comparable cytokine profiles can be observed in ventilated rats, lambs, and baboons [135–137]. Similar to MV, hyperoxia exposure is as an independent mediator of lung inflammation [112, 131, 138–140]. Hyperoxia induces inflammation primarily via increase in IL-1β as evidenced by experiments with overexpression and blocking of IL-1β signaling [141–144]. Moreover, some evidence exists for the role of *Csf1r*^+^ monocytes and macrophages in hyperoxia-induced lung injury [138]. Finally, oxidative stress induced by hyperoxia impacts the expression of large number of genes implicated in cell cycle, signal transduction, ECM turnover, coagulation, and alveolar growth [145]. The initial inflammatory response disturbs the homeostasis and triggers additional mediators and growth factors which in turn impact alveolar and microvascular formation [146, 147]. Depending on the model used however, the inflammatory response induced by hyperoxia might be considered moderate. To better reflect the clinical situation, various double-hit models combining postnatal hyperoxia with either pre- or postnatal LPS administration were developed [148–153]. Results of these studies vary depending on species, strain, and LPS dosage. Overall, both pre- and postnatal endotoxin amplifies the effects of hyperoxia exposure in dose-dependent manner [148, 152]. Additionally, only animals exposed to both hits developed prominent PH [152], as well as intense local and systemic inflammatory response [149, 151].

Both, MV and hyperoxia exposure induce alveolar hypoplasia in mice [154, 155], rats [156, 157], lambs [130], and baboons [136]. Ventilation in premature infants is associated with underdeveloped terminal airspace epithelium and epithelial apoptosis [158–160]. Parallel observations of epithelial inflammation and shedding, as well as epithelial hyperplasia were made in ventilated lambs [161, 162] and baboons [163] respectively. MV and hyperoxia exposure are further associated with increased [164, 165] or decreased [166, 167] alveolar epithelial type 2 (AT2) cells proliferation. Finally, both hyperoxia [168] and cyclic stretch [169] disrupt epithelial permeability in vitro.

Reduction in lung microvascularization is seen in BPD patients [170, 171] and in various animal models [172, 173] alike. Both, hyperoxia and ventilation inhibit pro-angiogenic VEGF signaling in rodent [172–174], rabbits [175], and baboons [176], reminiscent of observations in the lungs, plasma, and TAs of BPD patients [177–179]. Decreased levels of additional pro-angiogenic factors have also been observed. Expression levels of eNOS are decreased in hyperoxia-exposed mice and ventilated lambs [174, 180]. Lowered *Tie-2* and *Ang-1* levels are found in ventilated preterm infants [181] and *Ang-1* expression is decreased in hyperoxia-exposed mouse pups [182]. Similarly decreased in BPD patients and hyperoxia-exposed mice is the production of novel angiogenic markers such as *Foxf1* and *c-Kit* [183].

The mesenchymal damage in the “new” BPD manifests in form of thickened alveolar septa, defective ECM deposition, and interstitial fibrosis [147, 184, 185]. Comparable findings were made in various animal models, including ventilated lambs and baboons [136, 186], as well as ventilated and hyperoxia-exposed rodents [172, 187–189]. Among the most studied in context of late lung development and BPD are the FGF, TGF-β, and PDGFA signaling pathways. Among the FGF family, pulmonary FGF10 expression is decreased [190], while FGF2 TA levels are increased in BPD patients [191]. Elevated FGF2 expression is also found during compensatory lung growth in hyperoxia-exposed rats [192]. PDGFA, as well as its receptor PDGFRA, are critical for secondary septation and their loss results in decreased myofibroblasts migration and proliferation [193–195]. Decreased PDGFRA expression is found in animal hyperoxia BPD models [151, 194, 196] and the low expression of PDGFRA is associated with increased BPD incidence in male patients [197]. TGF-β signaling is essential for fetal lung development and is dynamically regulated during alveolarization [198, 199]. Increase in TGF-β expression was reported in TAs of BPD patients [200]. Similarly, an increased TGF-β expression is associated with alveolar hypoplasia secondary to hyperoxia, which could be prevented by treatment with TGF-β neutralizing antibody [201, 202]. Perturbances in both, PDGFA and TGF-β signaling are associated with defects in secondary septation, during which elastin is deposited at the top of the protruding septa by myofibroblast. This ECM scaffold provides a base for further alveolar formation [147]. Impaired elastic fibres formation and an increased expression of elastin and elastin or collagen cross-linking enzymes were noted in BPD patients [203–205], as well as ventilated and hyperoxia-exposed rodents and lambs [186–188, 205, 206].

It is important to note that considerable efforts have been made to establish less invasive therapies, avoiding intubation and mechanical ventilation. These strategies include sustained inflation approaches, such as nasal continuous positive airways pressure (NCPAP) or synchronised nasal intermittent positive pressure ventilation (SNIPPV) [207, 208]. Particularly the combination of NCPAP and early surfactant-replacement therapy is more effective in preventing BPD than continuous MV and elective surfactant replacement [207, 209]. However, meta-analysis revealed that it is the avoidance of endotracheal MV, rather than sustained inflations strategies themselves, which decreases the risk of BPD and death [208]. As even a brief intubation early in life can have severe consequences, less invasive methods of surfactant delivery, such as less invasive surfactant administration (LISA) and minimally invasive surfactant therapy (MIST), have recently been developed. In combination with NCPAP these strategies currently represent very promising approaches to decrease the BPD occurrence [208, 210–212].

## BPD phenotypes and endotypes

The multifactorial nature of BPD pathogenesis, combining various prenatal and postnatal insults results in several discrete endotypes and clinical phenotypes. As mentioned above, the biggest differences in clinical manifestation could be observed between the so-called “old”, profibrotic-like BPD phenotype and the “new” BPD characterized mainly by parenchymal and vascular damage [4, 5, 213]. Various classifications of BPD phenotypes have been proposed, including the categorization based on (i) severity [214], (ii) lung function (obstructive vs. restrictive phenotype) [215], or (iii) most effected tissue compartment [213, 216, 217]. Perhaps the most detailed classification, proposed in a recent review by Pierro et al., includes seven categories of BPD phenotypes: (i) parenchymal (characterized by alveolar simplification), (ii) peripheral airway (defined by bronchial hyperreactivity), (iii) central airway (stenosis, bronchomalacia, and tracheomalacia), (iv) interstitial (interstitial fibrosis and inflammation), (v) congestive (with pulmonary edema), (vi) vascular (dysmorphic vascularization and PH), and (vii) mixed phenotype [213].

Individual phenotypes do not only require different treatments but have different disease development and consequences in later life. The parenchymal phenotype, which is defined by arrest in alveolarization and decreased alveolar surface area, largely resembles emphysema. In fact, about two-thirds of BPD patients develop obstructive disease [215]. While the lung has capacity to continue alveolarization postnatally and parenchymal disease may improve over time, parenchymal damage in BPD has been previously associated with early onset chronic obstructive lung disease (COPD) in later life [213, 218]. The second obstructive phenotype—the peripheral airway phenotype, characterized by bronchoconstriction and hyperreactivity manifests similarly to asthma. However, studies have shown that at school age, these patients respond differently to treatments with β2-agonists than asthma patients and that the disease might be additionally characterized by structural changes in small airways [213, 216, 219].

In addition to BPD phenotypes, two main BPD endotypes are often recognized: (i) infection/inflammation (including chorioamnionitis) and (ii) placental dysfunction (including preeclampsia and IUGR) [213, 220]. While chorioamnionitis and infection represent the more common endotype, placental dysfunction in combination with IUGR constitute a more prominent background for vascular BPD phenotype [54, 221]. Ideally, in accordance with emerging personalized medicine approaches, distinct BPD phenotypes will be considered when assigning therapies and identifying possible complications in later life. However, details regarding underlying mechanisms and cellular integrations pertaining to individual phenotypes have not been yet deciphered.

## Novel approaches to study the preterm lung

### BPD in single-cell resolution

Understanding the lung composition on single-cell level has been a focus of substantial scientific research for more than a decade. During this period, more than 300 single-cell RNA sequencing (scRNA-seq) or single-nuclei RNA sequencing (snRNA-seq) human or animal lung datasets have been published. However, only a small fraction of these is dedicated to late lung development, and even fewer to the pathogenesis of BPD.

First exploratory scRNA-seq analysis of postnatal developing lung in mice were performed by Cohen et al., constructing a detailed single-cell map of the developing lung and lung progenitor cell populations covering the period from embryonic day (E)12.5 to postnatal day (P)7 [222]. The study identified 10 non-immune and 12 immune cell populations, revealing dynamic changes in population sizes, particularly during the pseudoglandular (E12.5) and canalicular stage (E18/19) of lung development. This data were further expanded in another study, characterizing lung immune (CD45^+^) cells between E18.5 and P21 [223]. When comparing the prenatal and postnatal immune cells authors identified a gradual increase in macrophage heterogeneity, as well as rapid increase in the proportion of lymphoid populations (2% *vs* 60% of all immune cells, respectively). Additional studies have focused on the developmental changes and postnatal adaptation in lung epithelial, endothelial, and stromal populations [224–227].

Several scRNA-seq studies have contributed to establishing a cell atlas of human fetal lung development [228–231]. No study to date has analysed lungs of BPD patients, although the scRNA-seq analysis of TA-derived cells to establish novel biomarkers and aid in stratifying BPD into endotypes has recently been proposed [232]. In contrast to the lack of studies in humans, few studies have explored the BPD pathogenesis at the single-cell level in the neonatal mouse hyperoxia model [151, 183, 226]. As mentioned above, the expression levels of angiogenic markers FOXF1 and c-KIT are decreased in the lungs of BPD patients [183]. This was confirmed by scRNA-seq in adult mouse lungs where hyperoxia exposure for first 7 days of life decreased the number of c-Kit^+^ endothelial cells (ECs) progenitors. Importantly, authors showed that adoptive transfer of c-Kit^+^ ECs improved lung angiogenesis and alveolarization in developing hyperoxia-exposed mice [183]. Substantial expression changes in all cell compartments were observed in the largest to date study of hyperoxia-exposed developing mice by Hurskainen et al., profiling over 66.000 lung cells at P3, P7, and P14 [151]. In this study, hyperoxia caused gradual changes in cell composition and expression patterns, particularly after 7 days of exposure. Within the stroma, authors identified transcriptomic shifts in myofibroblasts, pericytes, and *Col13a1*^+^ fibroblast, which were also among the most active signal senders and receivers in the hyperoxic lung. The study further revealed a substantial depletion of gCap (general capillary) cells and an increase in number of *Car4*^+^ aCap cells (aerocytes) after hyperoxia exposure [151]. The gCap cells were previously identified as putative distal lung vascular progenitors and regulators of capillary homeostasis, vasomotor tone, and repair [233]. The depletion of gCap ECs may contribute not only to the developmental injury, but also to the lack of repair capacity and an increased susceptibility to lung injury later in life, implying potential benefits of EC-derived cell therapies in BPD [151, 234]. In parallel, the *Car4*^+^ aCap cells showed pathological gene expression characterized by pro-inflammatory and anti-angiogenic markers. This is in agreement with recent reports, that aCap cells might contribute to septation [227] and revascularization following injury [235]. Moreover, the study highlighted the importance of inflammation in hyperoxia injury, with majority of the impacted transcriptional programs related to inflammatory response [151]. Finally, a recent study explored the long-term implication of neonatal hyperoxia [236]. Authors mapped lung cell populations in developing (P7) and adult (P60) mice previously exposed to hyperoxia for the first 3 days of life and identified persistent changes to AT2 subpopulations, predicting lasting perturbations to lung architecture and function [236].

While scRNA-seq studies so far have provided us with a somewhat complete map of the postnatally developing mouse lung, presently only a small portion of data derived from these studies have contributed to our understanding of BPD pathogenesis. As such, parallel studies in humans are still needed and further studies of BPD lungs are necessary to improve our interpretation of data obtained from animal studies. Finally, additional techniques, such as lineage tracing and spatial transcriptomics should be used to complement scRNA-seq to further investigate the role of newly identified cell populations, particularly rare cell subtypes and potential putative progenitors.

### Lung stem/progenitor cells—opportunities to regenerate the preterm lung

Lung constitutes a quiescent organ, with the turnover time gradually decreasing along the proximal–distal axis [237, 238]. Following injury, lung cells are typically activated by their microenvironment and directed to participate in remodelling or repair [237, 239, 240]. The same injurious stimuli can damage or inhibit stem cells' ability to differentiate, leading to decreased, incorrect, or inappropriately timed production of particular cell populations, contributing to the development of lung disease [241]. The role of stem cells in the dysplastic pulmonary growth, premature lung aging, and pathogenesis of BPD has previously been proposed [241]. However, while multiple populations of lung endothelial, epithelial, and stromal stem or progenitor cells have been described, relatively little is known about their role in late lung development or BPD [237]. Particularly of interest are questions why resident stem cells lose their function and whether it is more feasible to restore their progenitor potential, or rather supplement the injured lung with undamaged, exogenous, therapeutic stem cells.

Epithelial stem cells are the most studied putative progenitors in the lung [242–244]. These include proximal airways basal cells, secretory cells, bronchial alveolar stem cells (BASC), and distal AT2 cells. The fate of the progenitor basal cells, characterized by the expression of luminal cytokeratin KRT8, seems to be largely guided by the NOTCH signaling. Low levels of NOTCH expression predispose basal cells toward the secretory phenotype, while high levels lead to differentiation into goblet cells, and the absence of NOTCH results in the ciliated phenotype [245–249]. Although the progenitor capacity of BASC cells has been demonstrated in mice, their exact role in postnatal lung growth and even their existence in human lungs still remain controversial [237, 244, 250]. On the other hand, the role of distal AT2 cells in lung repair is well-established and has been broadly studied. Lineage-tracing and scRNA-seq studies shown, that (alveolar type) AT1 and AT2 cells originate from a common bipotent progenitor. In humans, the AT1/AT2 progenitors were reported in developing lungs at gestational week 15 [230]. Studies in mice suggest that the bipotent AT1/AT2 population splits into independent cell lines by E18.5 [251, 252]. AT2 cells were repeatedly shown to self-renew, differentiate into AT1 cells, and exhibit repair capacities even in matured lungs [253–256]. In regard to BPD pathogenesis, increased compensatory AT2-to-AT1 trans-differentiation was shown in the developing hyperoxia-exposed rats [257]. Early postnatal hyperoxia-exposure in mice resulted in reduced AT2 proliferation which persisted for up to 2 months [167]. Contradicting observations were however made in premature ventilated baboons, where AT2 hyperproliferation was observed [164], perhaps indicating that the nature, intensity, and timing of the injurious stimulus are critical in determining the way progenitor populations respond. Indeed, the molecular mechanisms involved in the AT2 progenitor capacity are largely unknown. Among the proposed pathways are the WTN, EGFR, and KRAS signaling pathways [256, 258]. Further, reports of progenitor-like AT1 cells [259] and the AT1-to-AT2 trans-differentiation also exist [260], and a specific *Hopx*^+^ AT1 population was shown to generate new AT2 cells in an adult mice post-pneumonectomy [261]. Finally, the progenitor role of so-called respiratory airway secretory cells (RAS) was also recently revealed [262]. RAS, which are located in human, but not mice proximal airways, differentiate exclusively into AT2 cells, a process regulated by NOTCH and WNT signalling. While this study explored the potential role of RAS in adult lung disease, future studies are needed to reveal their role in the neonatal lung.

In comparison to epithelial cells, less is known in regard to lung resident endothelial and mesenchymal stem cells. The rational for the search for endothelial progenitor cells (EPCs) is based in the hypothesis that the lung development is driven by pulmonary vessel formation [56, 263]. Numerous studies support the existence of resident EPCs in the postnatally developing lungs and a defective lung vascularization can be found in both, BPD patients and animal models of BPD [170, 172, 176, 177]. Moreover, inhibition of vessel formation in developing animals stunts lung development and results in alveolar hypoplasia [172, 264–266]. Importantly, pro-angiogenic interventions proved effective in improving lung alveolarization in animal BPD models [172, 267–269]. Reduction in number of resident and circulating EPCs was observed in murine BPD model [174], and the hyperoxia exposure decreased proliferation in human fetal lung endothelial colony-forming cells (ECFCs) in vitro [234]. Importantly, intravenously administered human cord blood-derived ECFCs were effective in restoring lung function, alveolar and vascular growth, and colony-formic capacity of resident ECFCs in hyperoxia-exposed developing mice [234]. Finally, some efforts have recently been made to identify markers of resident lung EPCs. Among the promising proposed candidates are above-mentioned markers FOXF1 and c-KIT, which expression is decreased in the lungs of hyperoxia-exposed rodents and BPD patients alike [151, 183].

The best described and most attractive among the somatic stem cells are mesenchymal stromal cells (MSCs), which can be easily isolated from bone marrow (BM-MSCs) or umbilical cord (UC-MSCs). Several studies have demonstrated the therapeutic properties of exogenous MSCs in experimental BPD, where UC- and BM-MSCs restored lung architecture and function, and attenuated inflammation and PH in developing rodents [270–274]. This evidence prompted further interest in MSC-based cell therapies for BPD and selected approaches are currently in early phase clinical trials [275–278]. Besides the cell-based therapies, MSC research further encompasses the study of cell-derived products, mainly extracellular vesicles (EVs). EVs represent a heterogenous population with smaller EVs (30–100 nm) also being referred to as exosomes [279]. Multiple studies have shown their role in cell communication during both, organ homeostasis and disease [280]. Human UC-MSC-derived EVs were shown to improve alveolar and vascular development, as well as lung function and RVH in hyperoxia-exposed developing mice [281–283]. Similar results were further observed in studies employing EVs from amniotic fluid-derived [284] and Wharton’s Jelly-derived MSCs [285]. Studies indicate that EVs exert their mostly anti-inflammatory effects by promoting an immunosuppressive CCR2-associated myeloid cell phenotype [283]. Similarly, antenatal delivery of BM-MSCs-derived EVs benefited rats with endotoxin-induced chorioamnionitis, resulting in reduced cytokine levels and improved lung growth and mechanics [286]. Finally, UC-MSCs-derived EVs protected lung architecture, vessel formation and inflammatory modulation in LPS-injected and mechanically ventilated developing mice [287].

In addition to exogenous MSCs, the notion of the resident lung MSC (L-MSC) population come from reports of MSCs in TAs from prematurely born infants, where their presence was identified as indicator of BPD morbidity and severity [288–290]. Resident L-MSCs were also described in human fetal lungs (gestational week 15–17) [291] and hyperoxia-exposed developing rodents [292, 293]. Hyperoxia exposure increased the number of L-MSCs and triggered expression of pro-inflammatory, pro-fibrotic, and anti-angiogenic genes [151, 293]. A scRNA-seq cell communication analysis revealed inflammatory signals from immune populations as main drivers of hyperoxia-induced changes in L-MSCs [293]. Importantly, hyperoxia-exposed human fetal L-MSCs exhibited decreased colony-forming capacity [291], while L-MSCs isolated from hyperoxia-exposed animals had decreased ability to support angiogenesis [292]. Although minimal criteria for MSCs characterization have been officially established [294], the definition remains rather crude and the identification of organ-specific MSCs, including the L-MSCs, lacks standardization. As a result, no L-MSC-specific marker has been accepted to date, although few markers have been proposed [292, 293, 295–297]. Notable among these is LY6A, also known as SCA-1 (stem cell antigen 1) [295, 297]. Recent studies have shown that L-MSCs may constitute a rather heterogenous population and their study might require more advanced methods, such as scRNA-seq or sc-proteomics [292, 293]. Another recently emerging candidate resident MSC population are the *Gli-1*^+^ repair-supportive mesenchymal cells [298, 299]. Progenitor properties of *Gli-1*^+^ cells were previously described in other organs, including bones [300, 301], teeth [300], and liver [302]. In the lung, *Gli-1*^+^ cells co-express *Acta2*, *Fgf10* and *Pdgfra*, thus resembling alveolar fibroblasts [225, 298]. In mice *Gli-1*^+^ MSCs were shown to aid epithelial regeneration following naphthalene-induced airway injury [298], and were shown to be increased in bleomycin-induced lung fibrosis in mice [303]. A more detailed characterization of all types of lung resident stem cell populations will clearly be of essence in understanding their role in normal and impaired lung development and regeneration.

## Conclusions

Great advancements in the understanding of BPD pathophysiology have been made since its first description almost 60 years ago. However, prevention and treatment of this multifactorial disease still pose major challenges. Moreover, most treatment strategies, however lifesaving, can contribute to the disease pathogenesis. Because BPD occurs in a still developing lung, there is a notable risk of life-long adverse effects. Additionally, due to the scarcity of human material, most of our current knowledge derives from experimental animal, mostly rodent models. Aberrant immune response, activation of pro-fibrotic and anti-angiogenic factors, as well as defects in alveolar and capillary formation are among the main features of BPD pathogenesis. More recent studies suggest additional roles in the development of BPD for maternal obesity, second-hand smoking, and pollution. However, no single animal model can fully replicate the complex nature of BPD. Therefore, how exactly do the prenatal and postnatal factors interrelate during the development of BPD, effect patients' recovery, and possibly contribute to the susceptibility to pulmonary diseases in later life remains unknown. Finally, the heterogenous nature of pulmonary cellular landscape represents a great challenge when identifying individual effector cells, particularly rare progenitors. Future novel multi-omics and interdisciplinary approaches will allow for more in-depth identification of rare cell types, cellular dynamics, and novel biomarkers. This knowledge will further enable development of more personalized therapeutics relevant to disease prevention, as well as acute and long-term organ repair. 

## Data Availability

Not applicable.

## Abbreviations

aCap: Aerocytes capillary cell
Ang-1: Angiotensin 1
AT1: Alveolar type 1 cell
AT2: Alveolar type 2 cell
BASCS: Bronchial alveolar stem cells
BM-MSC: Bone marrow-derived mesenchymal stromal cell
BPD: Bronchopulmonary dysplasia
Car4: Carbonic anhydrase 4
CD45: PTPRC, protein tyrosine phosphatase receptor type C
c-Kit: KIT proto-oncogene, receptor tyrosine kinase
Col1a1: Collagen 1a1
Col13a1: Collagen 13a1
COPD: Chronic pulmonary obstructive disease
Csf1r: Colony stimulating factor 1 receptor
E: Embryonic day
EC: Endothelial cell
ECM: Extracellular matrix
eNOS: Endothelial Nitric oxide synthase 3
EPC: Endothelial progenitor cell
EV: Extracellular vesicle
FGF: Fibroblast growth factor
FGF2: Fibroblast growth factor 2
FGF10: Fibroblast growth factor 10
FOXF1: Forkhead box F1
gCap: General capillary cell
GH: Growth hormone
HFD: High fat diet
Hopx: HOP homeobox
IGF-1: Insulin-like growth factor 1
IL-6: Interleukin 6
IL-8: Interleukin 8
IL-1β: Interleukin 1 beta
IUGR: Intrauterine growth restriction
KRT8: Keratin 8
LBW: Low body weight
LISA: Less invasive surfactant administration
L-MSC: Lung mesenchymal stromal cell
LPD: Low protein dietactin
LPS: Lipopolysaccharide
LY6A: Lymphocyte antigen 6 complex, locus A
MIST: Minimally invasive surfactant therapy
MSC: Mesenchymal stromal cell
MV: Mechanical ventilation
MVU: Maternal vascular underperfusion
NCPAP: Nasal continuous positive airways pressure
NICU: Neonatal intensive care unit
NOTCH: NOTCH protein
P: Postnatal day
PDA: Patent ductus arteriosus
PDGFA: Plateled-derived growth factor alpha
PDGFRA: Plateled-derived growth factor receptor alpha
PE: Preeclampsia
PH: Pulmonary hypertension
PVR: Pulmonary vascular resistance
RAS: Respiratory airway secretory cells
RVH: Right vascular hypertrophy
SCA1: Stem cell antigen 1
sc-proteomics: Single cell proteomics
scRNA-seq: Single cell RNA sequencing
sFlt-1: Soluble fms-like tyrosine kinase 1
SGA: Small for gestational age
SNIPPV: Nasal intermittent positive pressure ventilation
snRNA-seq: Single nuclear RNA sequencing
Stat5: Signal transducer and activator of transcription 5A
TA: Tracheal aspirate
TGF-β: Transcription growth factor beta
Tie-2: TEK receptor tyrosine kinase
TNF-α: Tumor necrosis factor alpha
UC-MSC: Umbilical cord-derived mesenchymal stromal cell
VEGF: Vascular endothelial growth factor
